# Distinct Metagenomic Signatures in the SARS-CoV-2 Infection

**DOI:** 10.3389/fcimb.2021.706970

**Published:** 2021-12-02

**Authors:** Yijun Liu, Hongyang Zhang, Xiaojun Tang, Xuejun Jiang, Xiaojuan Yan, Xizhao Liu, Jiang Gong, Kenley Mew, Hao Sun, Xiufeng Chen, Zhen Zou, Chengzhi Chen, Jingfu Qiu

**Affiliations:** ^1^ School of Public Health and Management, Chongqing Medical University, Chongqing, China; ^2^ Department of Nosocomial Infection, Chongqing Three Gorges Central Hospital, Chongqing, China; ^3^ Department of Clinical Laboratory, People’s Hospital of Wanzhou District, Chongqing, China; ^4^ School of Foreign Languages, Chongqing Medical University, Chongqing, China; ^5^ Department of Gastrointestinal Surgery, Chongqing Cancer Hospital, Chongqing, China; ^6^ Institute of Life Sciences, Chongqing Medical University, Chongqing, China

**Keywords:** SARS-CoV-2, COVID-19, gut microbiota, microbial community, metagenomic analysis

## Abstract

Severe acute respiratory syndrome coronavirus 2 (SARS-CoV-2) infection can cause gastrointestinal symptoms in the patients, but the role of gut microbiota in SARS-CoV-2 infection remains unclear. Thus, in this study, we aim to investigate whether SARS-CoV-2 infection affects the composition and function of gut microbiota. In this study, we demonstrated for the first time that significant shifts in microbiome composition and function were appeared in both SARS-CoV-2-infected asymptomatic and symptomatic cases. The relative abundance of *Candidatus_Saccharibacteria* was significantly increased, whereas the levels of *Fibrobacteres* was remarkably reduced in SARS-CoV-2-infected cases. There was one bacterial species, *Spirochaetes* displayed the difference between patients and asymptomatic cases. On the genus level, Tyzzerella was the key species that remarkably increased in both symptomatic and asymptomatic cases. Analyses of genome annotations further revealed SARS-CoV-2 infection resulted in the significant ‘functional dysbiosis’ of gut microbiota, including metabolic pathway, regulatory pathway and biosynthesis of secondary metabolites etc. We also identified potential metagenomic markers to discriminate SARS-CoV-2-infected symptomatic and asymptomatic cases from healthy controls. These findings together suggest gut microbiota is of possible etiological and diagnostic importance for SARS-CoV-2 infection.

## Introduction

An outbreak of coronavirus disease 2019 (COVID-19) caused by severe acute respiratory syndrome coronavirus 2 (SARS-CoV-2) has spread rapidly around the world (World Health Organization, 2021). Typical clinical symptoms of SARS-CoV-2 infection include fever, dry cough, fatigue, myalgia and dyspnea (Zhu et al., 2020). Gastrointestinal symptoms are underestimated symptoms of COVID-19, although more than 10% of patients are suffered from diarrhea, nausea and vomiting (Wang et al., 2020). Recently, one large clinical study from China demonstrated that up to 28.38% of COVID-19 patients with gastrointestinal symptoms did not have the typical respiratory manifestations (Jin et al., 2020). Similar results was also reported in Singapore, where 17% of patients with COVID-19 presented with diarrhea (Young et al., 2020). According to the latest reported investigations, the viral RNA was detectable in near 50% of patients’ stool samples (Ong et al., 2020; Pan et al., 2020; Xiao et al., 2020). These findings highlight the possibility of gastrointestinal tract as a major target organ of SARS-CoV-2 infection and suggest that gastrointestinal symptoms should not be disregarded for the virus infectivity, especially during the pandemic period.

Fecal-oral transmission is a potential route of SARS-CoV-2 infection. In April 2020, one single-cell transcriptomic analysis revealed that specific cell receptor ACE-II (ACE2) and transmembrane serine protease 2 (TMPRSS2), both of which were the key protein for entry of SARS-CoV-2 to the host cells, were highly expressed in the enterocytes of gastrointestinal tract (Zhang et al., 2020). It is theoretically plausible that some COVID-19 patients may present gastrointestinal symptoms. Intriguingly, the viral RNA was detectable and persisted positively in the feces for a long time, even when the throat swabs became negative (Wu et al., 2020; Xu et al., 2020). In our recent report, the distinctive clinical characteristics of an asymptomatic case of SARS-CoV-2 viral nucleotide detection was positive in anal swabs but negative in nasopharyngeal swabs for 42 days (Jiang et al., 2020). However, to date, there is no clear correlation of gastrointestinal symptoms and detectable viral RNA in the feces. Also, the pathophysiology of SARS-CoV-2-associated gastrointestinal symptoms remains unclear.

A vast diversity of gut microbiota colonizes in the human gastrointestinal tract that offers many physiological benefits to the host (Valdes et al., 2018). Disturbance of gut microbiota are frequently observed in patients with viral infections, such as hepatitis B virus (Ren et al., 2017), human immunodeficiency virus (Vázquez-Castellanos et al., 2018), influenza virus (Deriu et al., 2016), etc. On the one hand, virus may have substantial and intimate interactions with microbiota and is capable of disrupting the composition of gut microbiota, therefore leading to the increased inflammation detected in patients with gastrointestinal disorders (Ma et al., 2019). On the other hand, the altered gut microbiota probably elevates the susceptibility of host to virus infections, and presents overall more severe clinical symptoms (Hussain et al., 2018). The current available evidence together implies a potential role of gut microbiota in the progression and severity of SARS-CoV-2 infection.

A previous study suggested the potential value of the gut microbiota as a diagnostic biomarker and therapeutic target for COVID-19 (Gu et al., 2020). In addition, studies have also shown that fecal microbiota alterations were associated with fecal levels of SARS-CoV-2 and COVID-19 severity (Zuo et al., 2020). Devaux and collaborators reported that in COVID-19, the gastrointestinal dysbiosis is the consequence of a cascade of events that are found in most of the pathological processe (Devaux and Lagier, 2021). Compared with these investigations, our aim is to further identify potential metagenomic markers to discriminate SARS-CoV-2-infected symptomatic and asymptomatic cases from healthy controls. Therefore, herein, both the COVID-19 patients and asymptomatic infected cases were recruited to test whether SARS-CoV-2 infection was associated with alterations in the composition of gut microbiota. Our metagenomic analysis, for the first time, provides evidence that distinct metagenomic signatures are closely related with the asymptomatic and symptomatic SARS-CoV-2 infections. These findings will provide us new insights into the understanding of the etiology and potential mechanisms of COVID-19 in the gastrointestinal tract and are crucial for defining novel prevention and therapeutic strategies.

## Patients and Methods

### Study Design and Participants

A total of ten COVID-19 patients were recruited for study from Chongqing Three Gorges Central Hospital. All the patients were diagnosed with COVID-19 according to the Diagnosis and Treatment Protocol for Novel Coronavirus Pneumonia, released by the National Health Commission of China. The patients showed the gastrointestinal manifestations but did not have any other comorbidities (such as malignancy, hypertension, diabetes, cardiovascular diseases, chronic respiratory diseases, human immunodeficiency virus or hepatitis B virus infections, etc.) were enrolled. Ten asymptomatic SARS-CoV-2 infected cases were enrolled from Wanzhou People’s Hospital of Chongqing. Asymptomatic cases were identified to be infected with SARS-CoV-2 by using qRT-PCR assays on nasopharyngeal swabs. The asymptomatic cases did not show any clinical manifestations, including the abnormal chest CT images, decreased lymphocyte count and presence of symptoms on the day of testing and during the preceding 14 days. All the patients and asymptomatic cases were Wuhan residents, or had recently travelled to Wuhan, or the local resident who were the close contact of confirmed COVID-19 cases. Ten healthy control subjects were selected to represent an age- and sex- matched group with no health problems. Further inclusion criteria in the healthy control group included: no history of respiratory or gastrointestinal diseases, no history of virus infection, no smoking/drinking, no hypertension, no diabetes or any other kinds of chronic noncommunicable diseases, no use of antibiotics in the past 3 months and normal diet in the past 7 days. The total 30 participants were given instructions for providing fecal samples. The stool samples of patients were collected before the use of clinical drugs, the samples of asymptomatic cases and healthy controls were self-collected in fecal containers and stored at -80°C immediately. The female participants were instructed to not collect stool samples during their menstrual period. All blood samples were collected in the morning before eating breakfast and subjected to the clinical determination, including types and numbers of cells in blood, total protein, globulin, albumin, blood glucose, high-sensitivity C-reactive protein, D-Dimer, creatinine, alkaline phosphatase etc. This study was approved by the Ethics Committee in Chongqing Medical University. After receiving oral and written information, all the participants had signed the written informed consent.

### Stool Sample *DNA* Extraction

Total bacterial DNA of stool samples were extracted using the TIANamp Stool DNA Kit (Tiangen Biotech, Beijing, China; cat number: DP328) following the manufacturer’s instructions. The concentrations and purity of DNA were measured by a Turner TBS‐380 mini‐fluorometer (Turner Biosystems, Sunnyvale, CA, USA) and a NanoDrop™ 2000 spectrophotometer (Thermo Scientific, Wilmington, DE, USA). The extracted DNA was then stored at -20°C. The genomic DNA quality was further evaluated by 1% agarose gel electrophoresis before metagenomic sequencing.

### Library Construction and Metagenomic Sequencing

Paired-end libraries were prepared with a fragment average length of ~300 bp by using Covaris M220 (Covaris Inc., Woburn, MA, USA). Libraries were constructed by NEXTflex Rapid DNA-seq kit (Bioo Scientific Cor. Austin, TX, USA). Adapters that contained the full complement of sequencing primer hybridization sites were ligated to the blunt-end of fragments. All samples were sequenced in the Illumina HiSeq4000 instrument (Illumina Inc., San Diego, CA, USA) at Majorbio Bio-Pharm Technology Co., Ltd. (Shanghai, China).

### Data Quality Control

Sequencing adapter sequences were stripped from 3’ and 5’ paired end Illumina reads by the software of Seqprep (https://github.com/jstjohn/SeqPrep). Reads with lengths less than 50 bp, with a quality value less than 20, and with N bases were all removed by software of Fastp (https://github.com/OpenGene/fastp). The set of high-quality pair-end reads and single-end reads were used for further analysis. In addition, the reads that aligned to human genome (http://bio-bwa.sourceforge.net) and any hit associated or mated with the reads were removed. The metagenomic data were assembled by Megahit (https://github.com/voutcn/megahit), and the contigs with the length being or over 300 bp were selected. The obtained contigs were then used for the gene prediction and annotation.

### Gene Prediction, Taxonomy, and Functional Annotation

Each assembled contig was used to predict the open reading frames by MetaGene (http://metagene.cb.k.u-tokyo.ac.jp). The predicted open reading frames with lengths 100 bp or over were retrieved and translated into amino acid sequences by using the National Center for Biotechnology Information (NCBI) translation table (http://www.ncbi.nlm.nih.gov/Taxonomy/taxonomyhome.html/index.cgi?chapter=tgencodes#SG1). All the predicted genes were clustered by the software of CD-HIT (http://www.bioinformatics.org/cd-hit/) with the 95% sequence identity and 90% coverage. In each cluster, the longest sequences were selected as the representative sequences to construct non-redundant gene catalog. The set of high-quality reads were mapped to the representative sequences by using software of SOAPaligner with 95% identity (http://soap.genomics.org.cn/). The abundance of each sample was calculated and obtained. Subsequently, the representative sequences in the non-redundant gene catalog were aligned to NCBI non-redundant (NR) sequence database by Blastp (Version 2.2.28+, http://blast.ncbi.nlm.nih.gov/Blast.cgi) for taxonomic annotations. The representative sequences were blast with evolutionary genealogy of genes Non-supervised Orthologous Groups (eggNOG) database using Blastp for Cluster of orthologous groups of proteins (COG) annotations. The Kyoto Encyclopedia of Genes and Genomes (KEGG) annotations were performed by Blastp against KEGG database (http://www.genome.jp/keeg/). Genes were also annotated to Carbohydrate-active enzymes (CAZy). The annotations were carried out by Hmmscan (http://hmmer.janelia.org/search/hmmscan) with CAZy database (http://www.cazy.org) Version 5.0. Antibiotic resistance annotation was conducted using Blastp search (Version 2.2.28+) against ARDB database (http://ardb.cbcb.umd.edu). Virulent factor annotation was conducted by Blastp search (Version 2.2.28+) against VFDB database (http://www.mgc.ac.cn/VFs). The Comprehensive Antibiotic Resistance Database (CARD) was conducted by Blastp search (Version 2.2.28+) against CARD database (http://arpcard.Mcmaster.ca, Version 1.1.3). Gene Ontology (GO) annotation was conducted using blast2go (http://www.blast2go.com), against the GO database (http://www.geneontology.org). All the e-values were set as 1e-5.

### Statistical Analysis

Parametric one-way analysis of variance (ANOVA) or non-parametric Kruskal-Wallis H test was used to compare the significant differences among three groups. Fisher’s exact test or Chi-Square test was used to compare the differences on gender, contact history and enterotypes of the three groups. Kruskal-Wallis H test or Wilcoxon rank sum test was applied to determine differential abundance of metagenomic features. *Post hoc* test was carried out by using Turkey-Kramer test. Correlations were performed between blood indicators and metagenomic features using Spearman’s correlation. A *P*-value of <0.05 was considered statistically significant. Statistical analyses were performed using R software, version 3.6.0. or IBM SPSS Statistics, version 21.0.

## Results

### Taxonomic Characterization of Gut Microbiota

To determine whether gut microbiota is related with SARS-CoV-2 infections, the fecal samples of 10 symptomatic COVID-19 patients, 10 asymptomatic cases and 10 healthy controls were subjected to metagenomics sequencing. Of ten COVID-19 patients, the most common gastrointestinal manifestation was diarrhea (50%), followed by nausea (40%), anorexia (20%) and vomiting (20%). Most of the patients with gastrointestinal symptoms had fever (80%), but 70% did not have the dry cough and other respiratory symptoms. No significant differences were observed based on the age (*P*=0.147), gender (*P*=0.878) and contact history among subjects (*P*=0.211) in the three groups. The characteristics of participants were summarized in **Supplementary Table 1**.

A total of 3.1 billion 150 bp paired-end reads were generated with an average of 103.35 ± 14.32 million reads for each sample. After the quality control of sequence data, 3.06 billion high-quality reads, which were free of adaptor and human DNA contaminants, were obtained and used for assembly (details in the three groups were shown in the **Supplementary Table 2**). Reads were further assembled by Megahit software obtaining a total of 4,641,722 contigs. A total of 9,211,878 open reading frames were obtained from contigs by using metagene predicted program. In the constructed non-redundant gene catalog, 1,735,402 genes were included by using CD-HIT clustering program with a catalog average length of 736.05 bp (details shown in the **Supplementary Table 3**).

**Figure 1:**
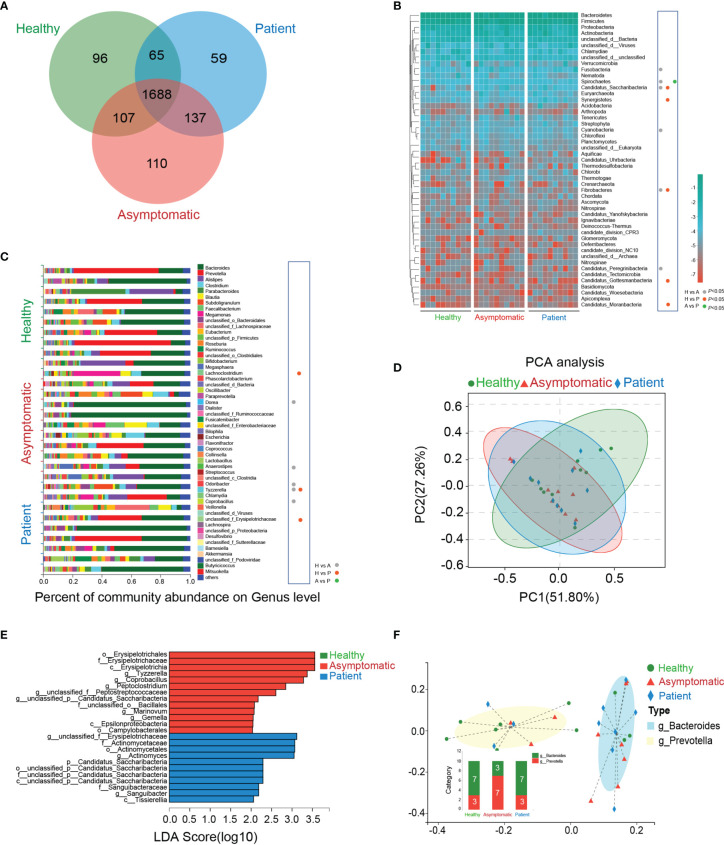
Taxonomic characterization of gut microbiota. **(A)** The unique and overlapped genera in the healthy controls, asymptomatic cases and COVID-19 patients were shown by Venn’s diagram (n=10). **(B)** Relative abundances of the top 50 gut microbiota on the phylum level in the three groups were depicted in the heatmap (n=10). H, healthy controls; A, asymptomatic cases; P, patients. **(C)** The percent of community abundance on the genus level in the three groups were shown (n=10, *P*<0.05, Kruskal-Wallis H test followed by Tukey-Kramer test) **(D)** Principal component analysis (PCA) and non-metric multidimensional scaling (NMDS) of microbial abundance on the genus level. **(E)** Linear discriminant analysis Effect Size (LEfSe) analysis on the genus level in the three groups. **(F)** Two enterotypes in the three groups based on the abundance of genera.

The Venn’s diagram displayed 96 unique genera in the healthy controls, 110 unique species in asymptomatic cases and 59 unique genera in the COVID-19 patients. Three groups shared the compositional overlap of the 1,688 genera in NR database (**Figure 1A**). According to the bacterial community profiles at the phylum level, the hierarchical heatmap of annotated species with NR database displayed the 50 most significant different species detected in all samples (**Figure 1B**). The abundance of *Fusobacteria* (*P*=0.017), *Spirochaetes* (*P*=0.038), *Candidatus_Saccharibacteria* (*P*=0.006), *Cyanobacteria* and *Candidatus_Peregrinibacteria* (*P*=0.008) in the asymptomatic cases were much higher than those of healthy controls, whereas the abundance of *Fibrobacteres* (*P*=0.045) was significantly reduced in the asymptomatic SARS-CoV-2 infected cases as compared with healthy controls. As compared with healthy controls, the abundance of *Candidatus_Saccharibacteria* (*P*=0.002), *Synergistetes* (*P*=0.045), *Candidatus_Gottesmanbacteria* (*P*=0.030) and *Candidatus_Moranbacteria* (*P*=0.007) in the confirmed COVID-19 patients were significantly increased, but the level of *Fibrobacteres* (*P*=0.031) was much lower than that of control group. The only difference on the abundance of bacterial species between patients and asymptomatic cases at phylum level was *Spirochaetes* (*P*=0.045).

As shown in the (**Figure 1C**), on the genus level, the abundance of *Dorea* (*P*=0.045), *Anaerostipes* (*P*=0.045), *Tyzzerella* (*P*=0.021) and *Coprobacillus* (*P*=0.009) in the healthy controls were sharply depleted in comparison to asymptomatic cases, while *Odoribacter* abundance in healthy controls was much higher than that of asymptomatic cases. As compared with healthy controls, the abundance of *Lachnoclostridium* (*P*=0.045), *unclassified_f_Erysipelotrichaceae* (*P*=0.021) and *Tyzzerella* (*P*=0.037) were all elevated in the patients’ stool samples. It is noteworthy that no significant abundance of bacterial species was found between asymptomatic and symptomatic cases. More importantly, in the genus level, there was only one kind of bacteria, *Tyzzerella*, changed significantly in both asymptomatic and symptomatic SARS-CoV-2 infected cases.

A principal component analysis (PCA) was carried out to test the extent of the similarity of the microbial communities in the three groups, respectively. The analysis indicated that the microbiota composition of the clusters was heterogeneous and significantly different among the three groups (**Figure 1D**). The linear discriminant analysis (LDA) distribution diagram analysis (LDA score>2.0) illustrated a clear alteration of the microbiota characterized by top four higher abundance of *o_Erysipelotrichales* (*P*=0.012)*, f_Erysipelotrichaceae* (*P*=0.012)*, c_Erysipelotrichia* (*P*=0.012)*, and g_Tyzzerella* (*P*=0.029) in patients and top four higher levels of *g_unclassified_f_Erysipelotrichaceae* (*P*=0.034), *f_Actinomycetaceae* (*P*=0.014), *o_Actinomycetales* (*P*=0.014) and *g_Actinomyces* (*P*=0.011) in asymptomatic cases (**Figure 1E**). The Calinski-Harabasz index indicated the optimal number of clusters was two. *g_Bacteroides* showed the relative high level of abundance in the enterotype 1, while the *g_Prevotella* contributed to enterotype 2 (**Figure 1F**). Taken together, the above analyses indicate SARS-CoV-2 infection shows dysbiosis in gut microecology.

### Function of the Gut Microbiota

**Figure 2:**
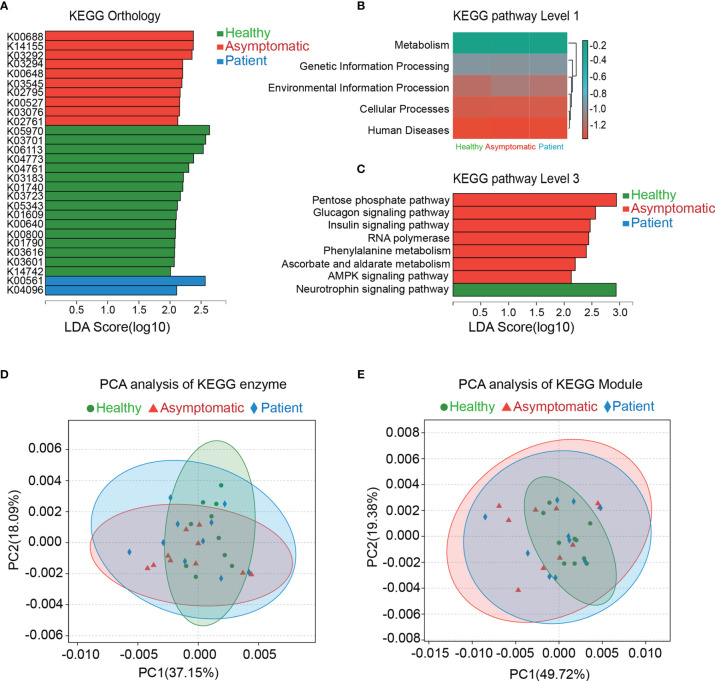
Functional analysis of gut microbiota. The genes were functional annotated to Kyoto Encyclopedia of Genes and Genomes (KEGG) database. Results of Linear discriminant analysis Effect Size (LEfSe) analysis on the KEGG orthology, KEGG pathway level 1 and level 3 were shown in the **(A–C)**. Principal component analysis (PCA) was carried out on the KEGG enzyme and module **(D, E)**.

The genes were functionally annotated to KEGG, COG, CAZy, ARDB, CARD and VFDB databases. The relative abundance values for each gene were calculated by counting the abundance of genes annotated to a feature. As shown in the **Figure 2**, the differences in microbial functions among three groups were observed on the KEGG pathways on the level 1, 3 and KEGG orthology (KO), enzyme and Module. In total, 28 KOs were changed significantly in the three groups, indicating that there were functional aspects of gut microbiota related with symptomatic of COVID-19 (**Figure 2A**). Thus, the KEGG network and pathway associations for the KOs were further used. Meanwhile, the KEGG pathways based on level 1 and level 3 were disrupted in the asymptomatic cases, relative to healthy controls and patients. For instance, pentose phosphate pathway (*P*=0.028), glucagon signaling pathway (*P*=0.025), insulin signaling pathway (*P*=0.006), RNA polymerase (*P*=0.040), phenylalanine metabolism (*P*=0.006), ascorbate and aldarate metabolism (*P*=0.010), AMPK signaling pathway (*P*=0.044) in asymptomatic group were all higher than those of healthy control group (**Figure 2B, C**). PCA based on KEGG enzymes and modules revealed slight differences in microbial functions among three groups (**Figures 2D, E**).

The eggNOG orthologous group of COVID-19 patients was partially altered based on the Linear Discriminant Analysis (LDA) effect size (LEfSe) by using Kruskal-Wallis test. The significant alterations of NOGs were COG3711 (*P*=0.047), COG3344 (*P*=0.022) and COG5283 (*P*=0.023) in the healthy controls, asymptomatic cases and patients, respectively (**Supplementary Figure 1**). However, no significant changes were found on the category and function among three groups. The CAZy results also revealed that the predicated functions of CAZy were also disrupted in the SARS-CoV-2 infected cases, including asymptomatic and symptomatic cases. The top significant CAZy on the family level were glycoside hydrolases (GH43_2) (*P*=0.041), glycosyl transferases (GT4) (*P*=0.012) and glycoside hydrolases (GH13_17) (*P*=0.026) in the health controls, asymptomatic cases and patients, respectively (**Supplementary Figure 2**).

To detect the distribution of antibiotic-resistant genes (ARGs) in the three groups, we compared high throughput sequencing reads of this study against the Antibiotic Resistance Database (ARDB) protein database. The overview of the ARDB results was shown in **Supplementary Figure 3**. As compared with healthy controls, the baca (Antibiotic type: bacitracin) and mls_mfs (Antibiotic type: macrolide) were remarkably decreased in all the SARS-CoV-2 infected cases (*P*=0.002 and *P*=0.009). There were significant differences on the types, antibiotic types and antibiotic resistance genes among three groups. To further verify these results, genes in the catalog were aligned against proteins in the Comprehensive Antibiotic Resistance Database (CARD) database. By using the software of Blastp, we identified 21 Antibiotic Resistance Ontology (ARO), which were significantly different in the three groups, including ARO:3003301 (*P*=0.022), ARO:3001293 (*P*=0.049) and ARO:3001301 (*P*=0.008) etc. (**Supplementary Figure 4**). In addition, virulence factors were relatively more abundant in stress protein in the SARS-CoV-2 infected cases on the VFDB level 2 (*P*=0.045). These altered virulence factors included cytolysin (*P*=0.006), MsrAB (*P*=0.043) and thioquinolobactin (*P*=0.040) (**Supplementary Figure 5**). Analysis based on the Gene Ontology (GO) database displayed the top 10 functions in the cellular component and molecular function displayed in a different manner, and the biological process did not show any difference among the three groups (**Supplementary Figure 6**). The analyses further indicate the disruption of gut microbiota function may play a potential role in the interaction effects between SARS-CoV-2 infection and gastrointestinal symptoms produced by the dysbiosis of gut microbiota.

### Relationship Between Gut Microbiota and Function

**Figure 3:**
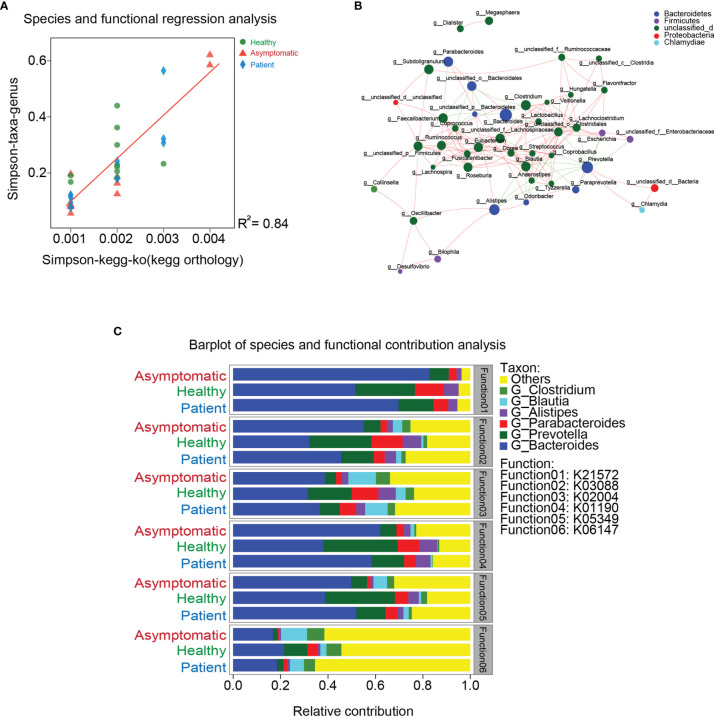
Relationship between gut microbiota and function. **(A)** The linear regression analysis was used to test the relationships between gut microbiota and their functions. **(B)** Co-occurrence network of bacteria from each group was constructed on the genus level based on the Spearman analysis. **(C)** Co-occurrence networks with scattered genera from six primary phyla.

To investigate whether the functions of gut microbiota are associated with their compositions, the linear regression analysis was carried out. As shown in **Figure 3A**, the coefficient of determination (R2) was higher than 0.8, indicating similarity of gut microbiota composition was significantly correlated with microbial functions. These results also suggest disruption of microbiota can further lead to the dysfunctions of gut microbiome. To describe the potential relationships occurring among species within the communities of gut microbiota, the co-occurrence network of bacteria from each group was constructed on the genus level based on the Spearman analysis (**Figure 3B**). The three groups mainly feature co-occurrence networks with scattered genera from six primary phyla, including *Bacteroidetes*, *Firmicutes*, *Proteobacteria*, *Actinobacteria*, *Chlamydiae* and *unclassified_d* (**Figure 3C**). The potential relationships between COG, CAZy, CARD, ARDB, VFDB, GO function and gut microbiota were also visualized in the **Supplementary Figure 7**. Additionally, the main contributors of species to the functions of COG, CAZy, CARD, ARDB and VFDB were shown in the **Supplementary Figure 8**.

### Gut Microbiota-Based Prediction

**Figure 4:**
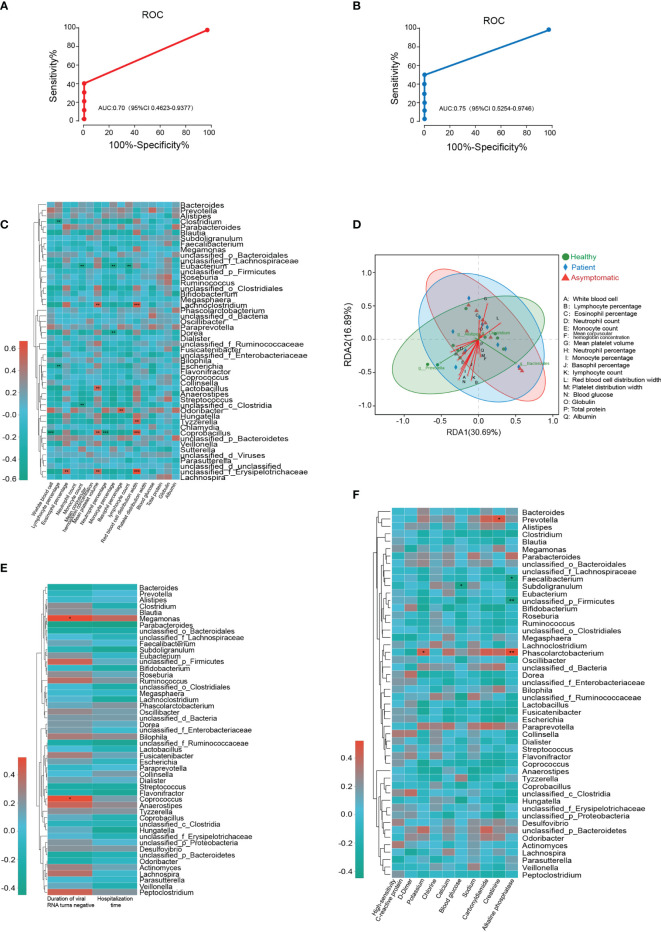
Gut microbiota-based prediction and their association with clinical indices. **(A, B)** Receiver operating characteristic (ROC) analysis of *Tyzzerella* for its ability to detect asymptomatic cases and patients. **(C)** Microbial species correlated with clinical indices of all the participants based on Spearman correlation analysis. **(D)** Relationships among clinical indices and top four gut bacterial were analyzed by using redundancy analysis (RDA) **(E)** Microbial species correlated with duration of viral RNA turns negative and hospitalization time. **(F)** Microbial species correlated with high-sensitivity C-reactive protein, D-Dimer, potassium, chlorine, calcium, blood glucose, sodium, carbonyldiamine, creatinine, alkaline phosphatase.

To investigate the potential diagnostic value of bacterial taxonomic biomarkers in the gut microbiome for distinguishing the symptomatic SARS-CoV-2 infection, a random forest model was built. The 30 most species identified by the random forest model were depicted in rank order of contribution to their prediction accuracy, including *g_Gemella*, *g_Dermacoccus*, *g_Terribacillus* and *g_Sneathia* etc. (shown in **Supplementary Figure 9**). Next, the interpolated area under the receiver operating characteristic (ROC) curves (AUC) was calculated for the classifier based on the ten-fold cross-validation results. Unfortunately, symptomatic nor asymptomatic cases were not able to be classified successfully, with a low AUC of 0.65 (95% confidence interval, CI: 0.40-0.87) and 0.62 (95% CI: 0.36-0.86) (**Supplementary Figure 9**). Given that *Tyzzerella* was the key species changed in both asymptomatic and symptomatic SARS-CoV-2 infected cases. The ability of *Tyzzerella* was tested to detect asymptomatic cases and patients. We successfully classified SARS-CoV-2 infected cases with a relatively high classification. The AUC was 0.70 (95% CI 0.46-0.93) for asymptomatic cases and 0.75 (95% CI 0.55-0.97) for patients, respectively (**Figures 4A, B**).

### Association Between Gut Microbiota and Clinical Indices

Next, we aimed to test whether the microbial species correlated with clinical indices of all the participants based on Spearman correlation analysis. As illustrated in the **Figure 4C**, two gut microbiotas on the genus level, *Clostridium* and *Escherichia* negatively correlated with the levels of lymphocyte percentage, whereas *unclassified_ f_Erysipelotrichaceae* was highly positively correlated with eosinophil percentage, mean platelet volume and red blood cell distribution width. *Tyzzerella*, *Lachnoclostridium* and *Coprobacillus* were also positively correlated with the level of red blood cell distribution width. Two another species *unclassified_c_Clostridia* and *Eubacterium* were negatively correlated with the monocyte count, while the monocyte percentage was related with the alterations of *Dorea* and *Eubacterium* abundance. In addition, white blood cell was negatively correlated with *Coprobacillus* and basophil percentage was positively related with *Odoribacter*. Neutrophil percentage and lymphocyte count were only correlated with the abundance of *Eubacterium* and *Coprobacillus*, respectively. The relationships among clinical indices and top four gut bacterial were analyzed by using redundancy analysis (RDA), and the results were shown in **Figure 4D**. To further determine the relationship between microbiota and clinical outcome of SARS-CoV-2 infected cases, the Spearman correlation analysis was conducted. The results illustrated the abundance of Megamonas and Coprococcus were positively correlated with the duration of virus RNA turns negative for the first time. However, the heatmap results demonstrated no significant correlation was observed between microbial species and hospitalization time (**Figure 4E**). In SARS-CoV-2 infected cases, our results also revealed both potassium and alkaline phosphatase levels were positively correlated with the abundance of *Phascolarctobacterium*. *Provotella* was positively correlated with the level of creatinine, while the blood glucose was negatively correlated with the abundance of *Subdoligranulum*. In addition, alkaline phosphatase levels were negatively correlated with two species of gut microbiota, *Faecalibacterium* and *unclassified_p_Firmicutes* (**Figure 4F**).

## Discussion

In this study, by using high-throughput metagenomic sequencing, the gut microbiota was analyzed in terms of taxonomic profiles, genetic functions, and associations with clinical indices of SARS-CoV-2 infected cases and healthy controls. Herein, we identified several compositional and functional alterations of the gut metagenome that may be related to SARS-CoV-2 infection. This study not only highlights gut metagenomic markers are able to differentiate between SARS-CoV-2-infected cases and healthy controls with a relative high level of specificity, but also raises the possibility for a complementary approach for potential treatment of COVID-19.

Currently, emerging studies demonstrate gastrointestinal infection is one of the clinical presentations of COVID-19, because the convincing evidence showing the high expression of ACE2 in intestinal enterocytes and positive viral RNA in the rectal swabs (Jiang et al., 2020; Lamers et al., 2020). Our results further indicated that SARS-CoV-2 infected cases had only a moderate degree gut bacterial dysbiosis. These findings are able to partially explain the presence of gastrointestinal symptoms of COVID-19, since disturbance of gut microecology mostly results in the gastrointestinal disorders (Simrén et al., 2013). Here, only two species, *Candidatus_Saccharibacteria* and *Fibrobacteres*, both of which changed markedly in SARS-CoV-2 infected cases, were identified on the phylum level. These results were consistent with reports of *Fibrobacteres* being associated with inflammatory bowel disease. Importantly, we identified the key species *Tyzzerella* on the genus level was remarkably increased in both symptomatic and asymptomatic cases. Such changes in the abundance of *Tyzzerella* have also recently been reported for irritable bowel syndrome. Moreover, the significant changes of *Tyzzerella* were highly associated with chronic intestinal inflammation (Chen et al., 2018). *Tyzzerella* was also identified significantly associated with high psychoneurological symptom cluster and lifetime cardiovascular disease risk profile (Kelly et al., 2016; Bai et al., 2020). On the other hand, high-sugar diet and high−lipid diet rats have lower concentration of *Tyzzerella* after treatmen (Yue et al., 2019; Zhang et al., 2020). Besides, *Tyzzerella* nexilis were first found that could encode the α-N-acetylgalactosaminidase and β-N-acetylhexosaminidase genes (Kelly et al., 2016; Bai et al., 2020). However, the specific roles of *Tyzzerella* are unclear for now. Of note, we observed the AUC of *Tyzzerella* for distinguishing the asymptomatic, symptomatic cases from healthy controls were 70% and 75%, respectively. The acceptable AUC values may imply the potential diagnostic value of *Tyzzerella* as the bacterial taxonomic biomarker for identification of SARS-CoV-2 infection. However, we did not observe the significant relation between *Tyzzerella* and clinical outcome, despite the *Tyzzerella* abundance was positively correlated with the level of red blood cell distribution width. This data suggests the lack of association of *Tyzzerella* and disease prognosis, which may be confounded by many factors, such as individual sensitivity and heterogeneity.

Our analysis of bacterial gene functions indicating there may be a ‘functional dysbiosis’, rather than a specific microbial species that has a direct association with pathophysiology of SARS-CoV-2 gastrointestinal infection. Intriguingly, the SARS-CoV-2-infected cases displayed the distinct functions of gut microbiota as compared with healthy controls. Moreover, the asymptomatic and symptomatic cases also showed the several differences on the functional alterations of gut microbiome. For instance, the metagenomes of COVID-19 patients were specially enriched in genes associated with puromycin biosynthesis, which indicated that increased puromycin may contribute to symptomatic COVID-19 by blocking the synthesis of protein (Marciano et al., 2018). In both asymptomatic cases and patients, we observed the enriched levels of genes associated with RNA polymerase. Indeed, the RNA polymerase is necessary for SARS coronavirus to replicate in infected cells (Gao et al., 2020). Moreover, the genes related to the RNA degradation were specifically enriched in the asymptomatic cases, that may indicate the degradation of genome RNA has a potential positive benefit during SARS-CoV-2 infection. This may also partially explain the asymptomatic clinical characteristics of participants. It is also worth noting, the genes of healthy controls mostly enriched in the ribosome. Since ribosomes are micro-machines for making proteins in the cell, impaired ribosomal function may imply disruption of protein synthesis in SARS-CoV-2 infection (Lafontaine and Tollervey, 2001). Together, our results propose the possibility that specific functions of gut microbiota may be linked with symptoms or progression of specific disease, including COVID-19. Even though these findings cannot provide direct evidence for casual effects, by indicating that gut metagenome may play a vital role in the progression of SARS-CoV-2 infection. Furthermore, alterations of gut microbiota composition or function induced by SARS-CoV-2 infection may itself play a role in increasing the susceptibility to other kinds of diseases.

This study has several important strengths. First, both the symptomatic patients and asymptomatic SARS-CoV-2 infected-cases were recruited. This design provides a unique opportunity to examine whether the alteration of microbiota is associated with the occurrence of symptoms in the gastrointestinal tract. Second, the clinical outcome and indices were carefully collected and the relationships between microbiome and clinical indices were also evaluated. Furthermore, stool specimens were collected according to a stringent protocol, with metagenomic sequencing and downstream analyses following the strict data quality control (Barbara et al., 2012). However, the certain limitations should also be considered. Firstly, a relatively small number of participants were included in this study because of the precise inclusion and exclusion criteria and the relative low incidence of gastrointestinal symptoms in the COVID-19 patients. This small size of sample may have limited the statistical power to identify the other vital microbiota or biological pathways that may influence SARS-CoV-2 infection. Secondly, the gut microbiota can be influence by some major disturbing factor, such as alcohol use, smoking and dietary habit (Wang et al., 2016). Although the enrolled subjects did not have the history of smoking or drinking, the dietary habit became the major uncontrolled variable. Thirdly, there was a lack of similar clinical investigations for comparison. Therefore, the prospective studies are warranted to verify whether the presence of identified microbiota precede severity and progression of SARS-CoV-2 infection.

There are several limitations of this study that should be mentioned. First, the sample size of this study is relatively small. All the samples from patients and asymptomatic cases were collected during the first outbreak time of COVID-19 in Chongqing, China. The small sample size and unproperly controlled cofounders might mainly occur due to the strict inclusion criteria. Second, the design of this study was a single-center, cross-sectional study, we did not follow individuals up over time, and the temporal link between the outcome and the exposure cannot be determined. The major strength is that the potential correlations between clinical variables and gut microbiota were identified by using metagenomic method. These findings will provide us an overview of gut microbiota in COVID-19 patients, asymptomatic cases and healthy volunteers.

In summary, this study provides a novel insight that gut microbiota may be involved in the infection of SARS-CoV-2. Although it is not clear whether alterations of gut microbiota composition and their functions directly cause grastrointestinal symptoms of COVID-19, structural and functional shifts in the microbiota may further lead to disruption of intestinal microenvironment integrity. The present study also highlights that the progression or serevity of SARS-CoV-2 infection may be influenced by the metabolic output of the entire microbiota. However, further investigations uncovering the prospective relation of the microbiome with SARS-CoV-2 infections are still needed.

## Data Availability Statement

The datasets presented in this study can be found in online repositories. The names of the repository/repositories and accession number(s) can be found below: https://www.ncbi.nlm.nih.gov/, PRJNA762232. The data presented in this study were deposited in the SRA repository, accession number: SAMN21380773-SAMN21380775.

## Ethics Statement

The studies involving human participants were reviewed and approved by ethics committee of Chongqing Medical University. The patients/participants provided their written informed consent to participate in this study.

## Author Contributions

Conceptualization was provided by CC and JQ. The original draft of the manuscript was written by YL and HZ. Funding acquisition was performed by JQ and ZZ. The methodology was developed by CC, XJ and ZZ. Investigations were carried out by XY, XT and XL. Resources were provided by JG, XY, XL and JQ. HS, KM and XC provided supervision and revised the manuscript. All authors contributed to the article and approved the submitted version.

## Funding

This work was supported by Emergency Task-Force Project on the Prevention and Control of Novel Coronavirus of Chongqing Municipal Education Commission (KYYJ202005); Natural Science Foundation of Chongqing (cstc2020jcyj-zdxmX0029 and cstc2020jcyj-msxmX0155).

## Conflict of Interest

The authors declare that the research was conducted in the absence of any commercial or financial relationships that could be construed as a potential conflict of interest.

## Publisher’s Note

All claims expressed in this article are solely those of the authors and do not necessarily represent those of their affiliated organizations, or those of the publisher, the editors and the reviewers. Any product that may be evaluated in this article, or claim that may be made by its manufacturer, is not guaranteed or endorsed by the publisher.

## Supplementary Material

Supplementary Figure 1
**(A)** Linear discriminant analysis Effect Size (LEfSe) analysis on the Cluster of orthologous groups of proteins (COG)-Non-supervised Orthologous Groups (NOG) level. **(B, C)** Heatmaps of COG category and function in the three groups.

Supplementary Figure 2
**(A)** Heatmaps of carbohydrate-active enzymes (CAZy) on the class level. **(B)** Linear discriminant analysis Effect Size (LEfSe) analysis on CAZy on the family level.

Supplementary Figure 3
**(A)** Percent of Antibiotic Resistance Genes Database (ARDB) on the class level. **(B)** Linear discriminant analysis Effect Size (LEfSe) analysis in ARDB on the type level. **(C, D)** LEfSe analysis in ARDB on the antibiotic type level and antibiotic resistance gene level.

Supplementary Figure 4
**(A)** Heatmaps of Comprehensive Antibiotic Resistance Database (CARD) on the class level. **(B)** Linear discriminant analysis Effect Size (LEfSe) analysis in CARD on the Antibiotic Resistance Ontology (ARO) level.

Supplementary Figure 5
**(A, B)** Heatmaps of virulent factor database (VFDB) on the level 1 and level 2. H, healthy controls; A, asymptomatic cases: P, patients. **(C)** Linear discriminant analysis Effect Size (LEfSe) analysis in VFDB on the virulence factors.

Supplementary Figure 6
**(A–C)** Analysis based on the Gene Ontology (GO) database displayed the top 10 functions in the biological process, cellular component and molecular function in the three groups.

Supplementary Figure 7
**(A)** The potential relationship between cluster of orthologous groups of proteins (COG) and gut microbiota. **(B)** The potential relationship between virulent factors and gut microbiota. **(C)** The potential relationship between antibiotic resistance ontology (ARO) and gut microbiota. **(D)** The potential relationship between antibiotic resistance genes (ARG) and gut microbiota. **(E)** The potential relationship between carbohydrate-active enzymes (CAZy) and gut microbiota.

Supplementary Figure 8
**(A)** Main contributors of species to the functions of cluster of orthologous groups of proteins (COG). **(B)** Main contributors of species to the functions of carbohydrate-active enzymes (CAZy). **(C)** Main contributors of species to the functions of antibiotic resistance genes (ARG). **(D)** Main contributors of species to the functions of antibiotic resistance ontology (ARO). **(E)** Main contributors of species to the functions of virulent factors.

Supplementary Figure 9
**(A)** The 30 most species identified by the random forest model were shown in rank order of contribution to their prediction accuracy. **(B, C)** Receiver operating characteristic curves of symptomatic nor asymptomatic cases were not able to be classified successfully.
