# Supplementary figures and images for: Distinct Metagenomic Signatures in the SARS-CoV-2 Infection
Source: Front Cell Infect Microbiol. 2021 Dec 2;11:706970. doi: 10.3389/fcimb.2021.706970 (PMC8674698; doi:10.3389/fcimb.2021.706970)

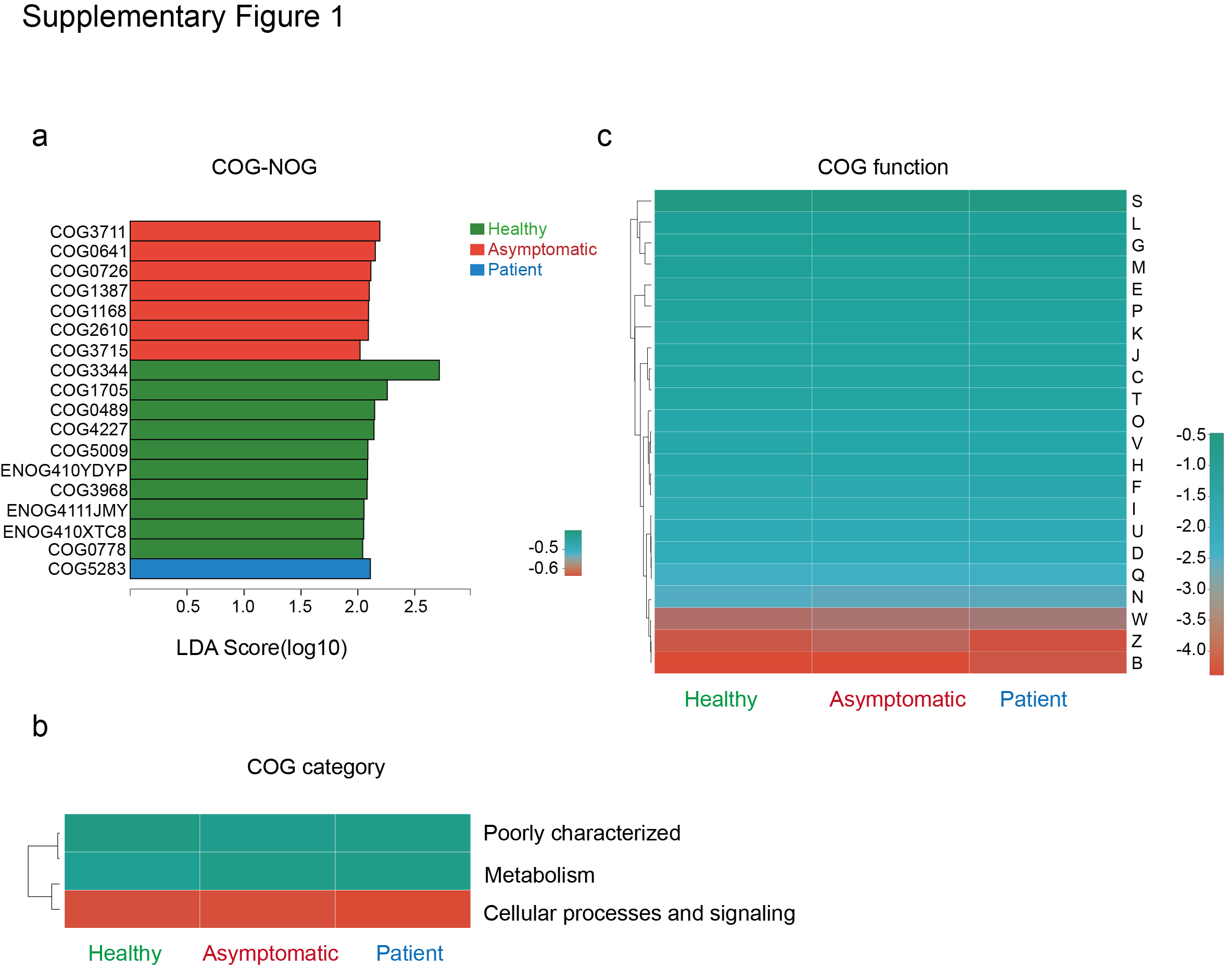

Supplement: Supplementary Figure 1 — (A) Linear discriminant analysis Effect Size (LEfSe) analysis on the Cluster of orthologous groups of proteins (COG)-Non-supervised Orthologous Groups (NOG) level. (B, C) Heatmaps of COG category and function in the three groups. [file Image_1.jpeg]

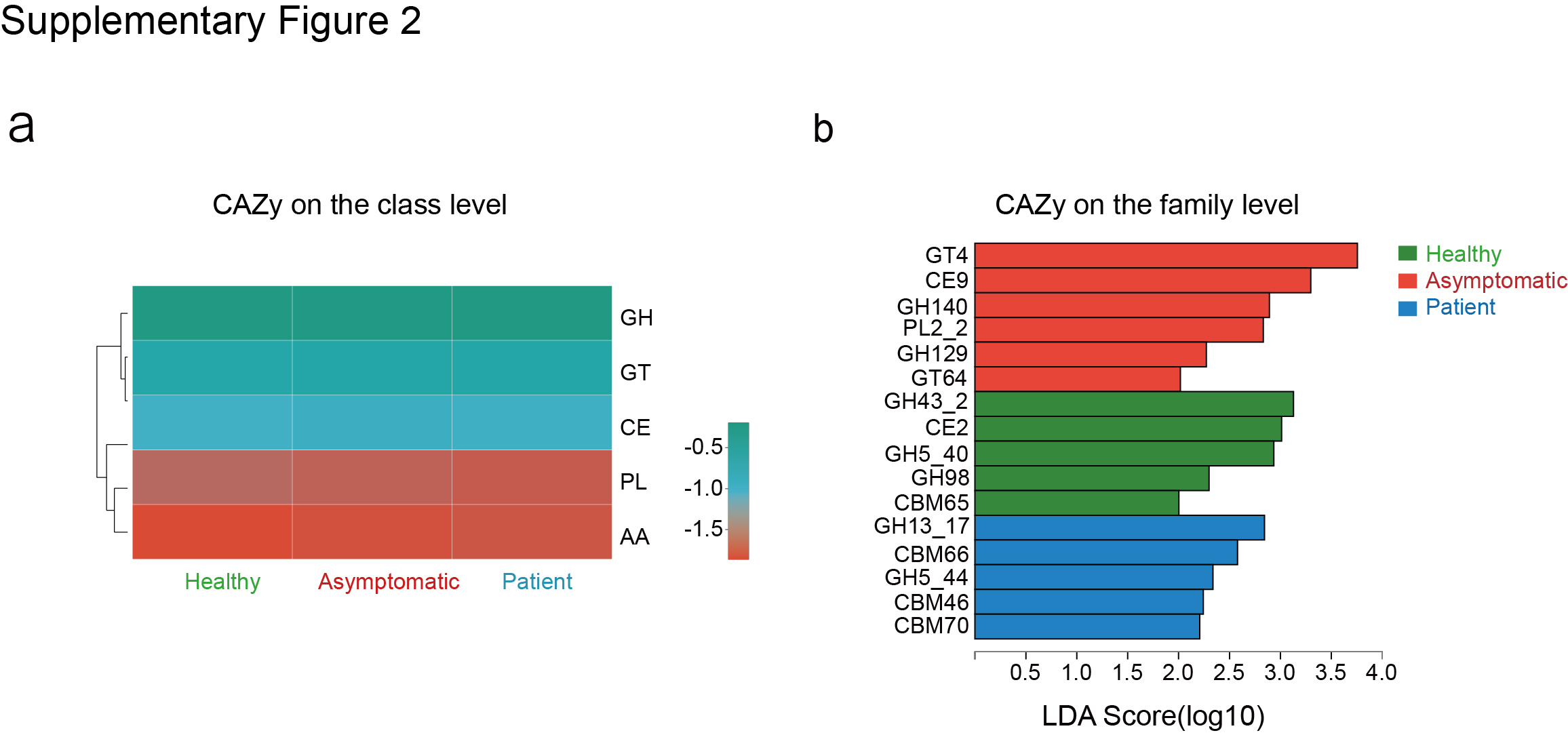

Supplement: Supplementary Figure 2 — (A) Heatmaps of carbohydrate-active enzymes (CAZy) on the class level. (B) Linear discriminant analysis Effect Size (LEfSe) analysis on CAZy on the family level. [file Image_2.jpeg]

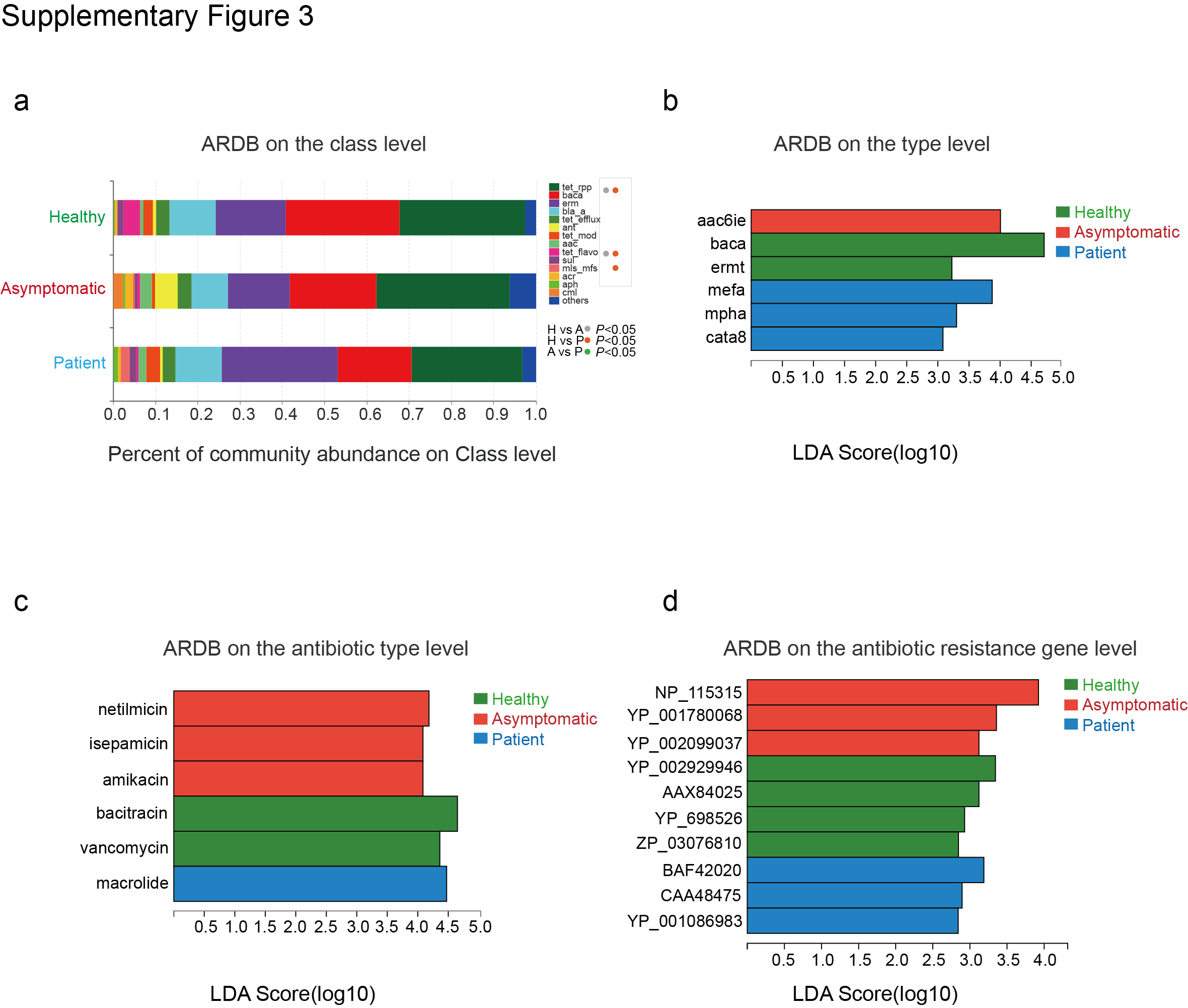

Supplement: Supplementary Figure 3 — (A) Percent of Antibiotic Resistance Genes Database (ARDB) on the class level. (B) Linear discriminant analysis Effect Size (LEfSe) analysis in ARDB on the type level. (C, D) LEfSe analysis in ARDB on the antibiotic type level and antibiotic resistance gene level. [file Image_3.jpeg]

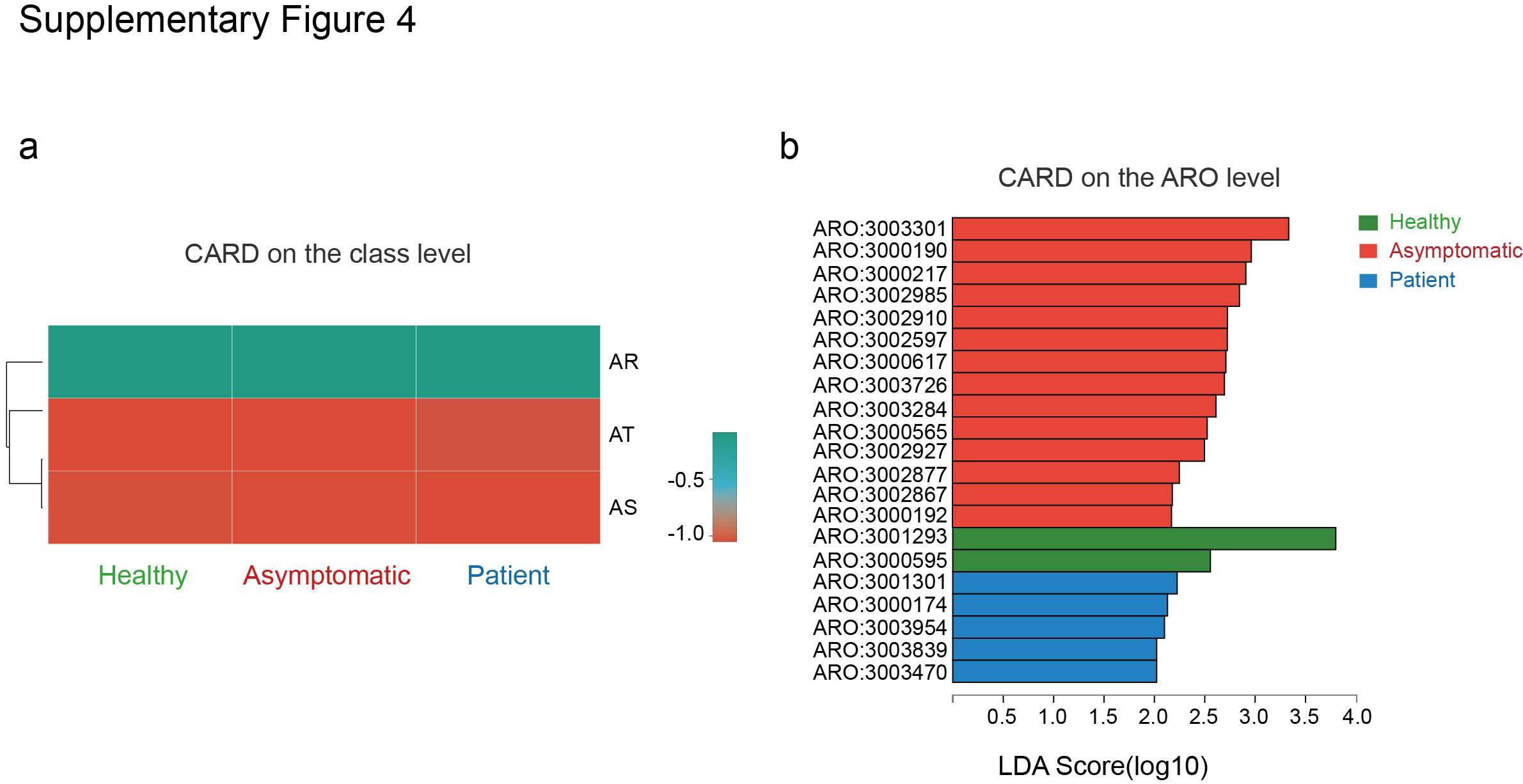

Supplement: Supplementary Figure 4 — (A) Heatmaps of Comprehensive Antibiotic Resistance Database (CARD) on the class level. (B) Linear discriminant analysis Effect Size (LEfSe) analysis in CARD on the Antibiotic Resistance Ontology (ARO) level. [file Image_4.jpeg]

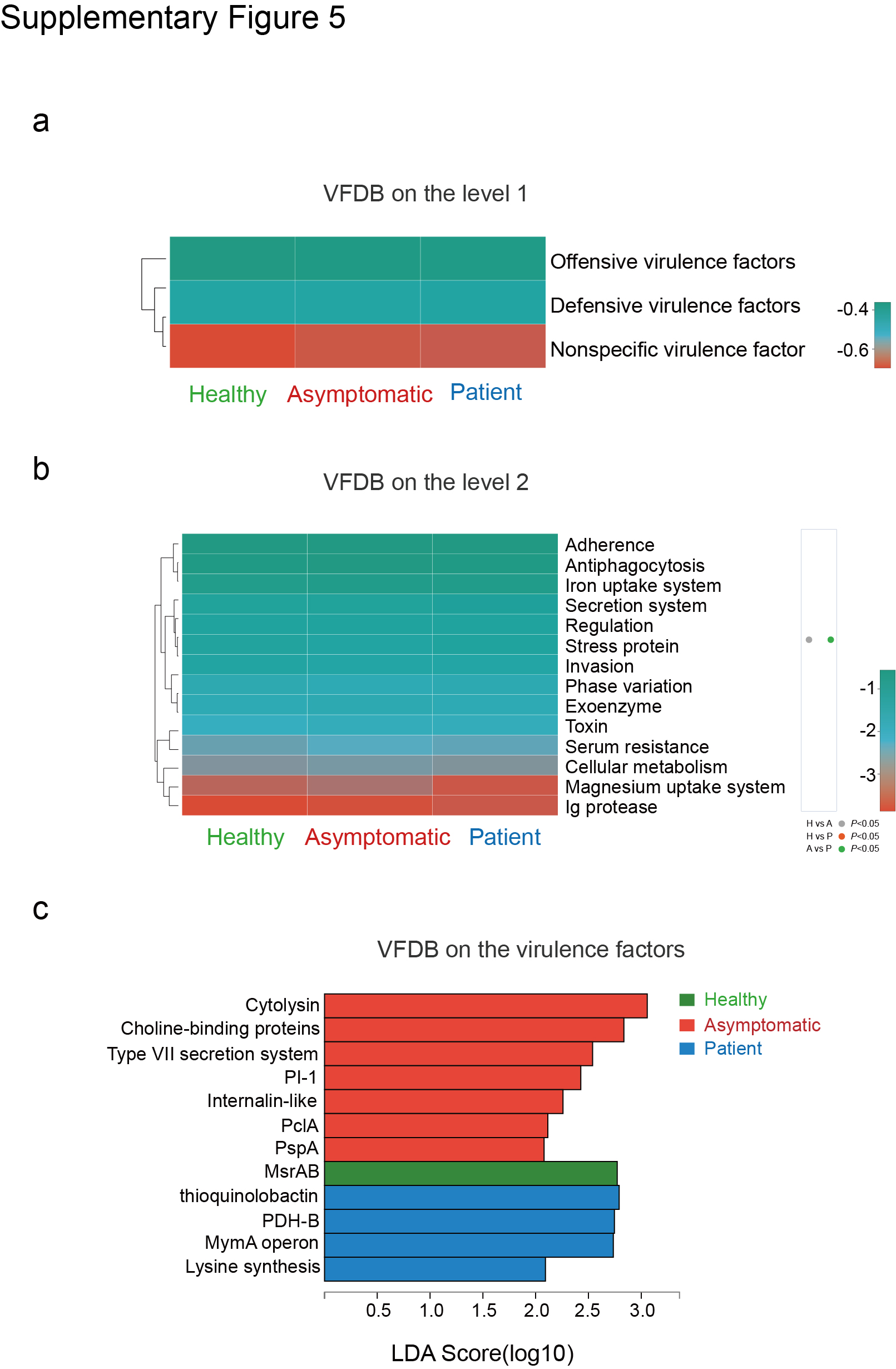

Supplement: Supplementary Figure 5 — (A, B) Heatmaps of virulent factor database (VFDB) on the level 1 and level 2. H, healthy controls; A, asymptomatic cases: P, patients. (C) Linear discriminant analysis Effect Size (LEfSe) analysis in VFDB on the virulence factors. [file Image_5.jpeg]

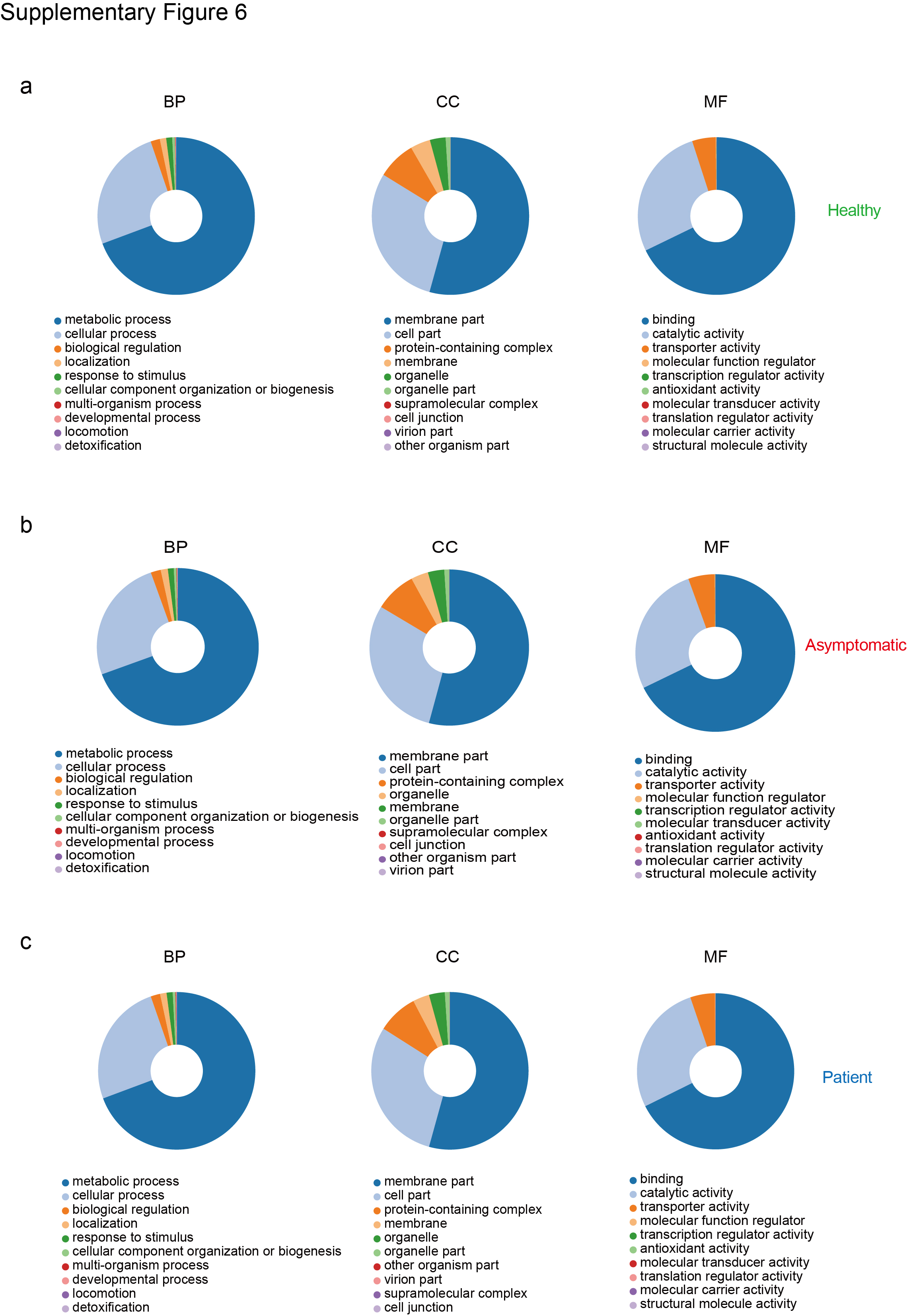

Supplement: Supplementary Figure 6 — (A–C) Analysis based on the Gene Ontology (GO) database displayed the top 10 functions in the biological process, cellular component and molecular function in the three groups. [file Image_6.jpeg]

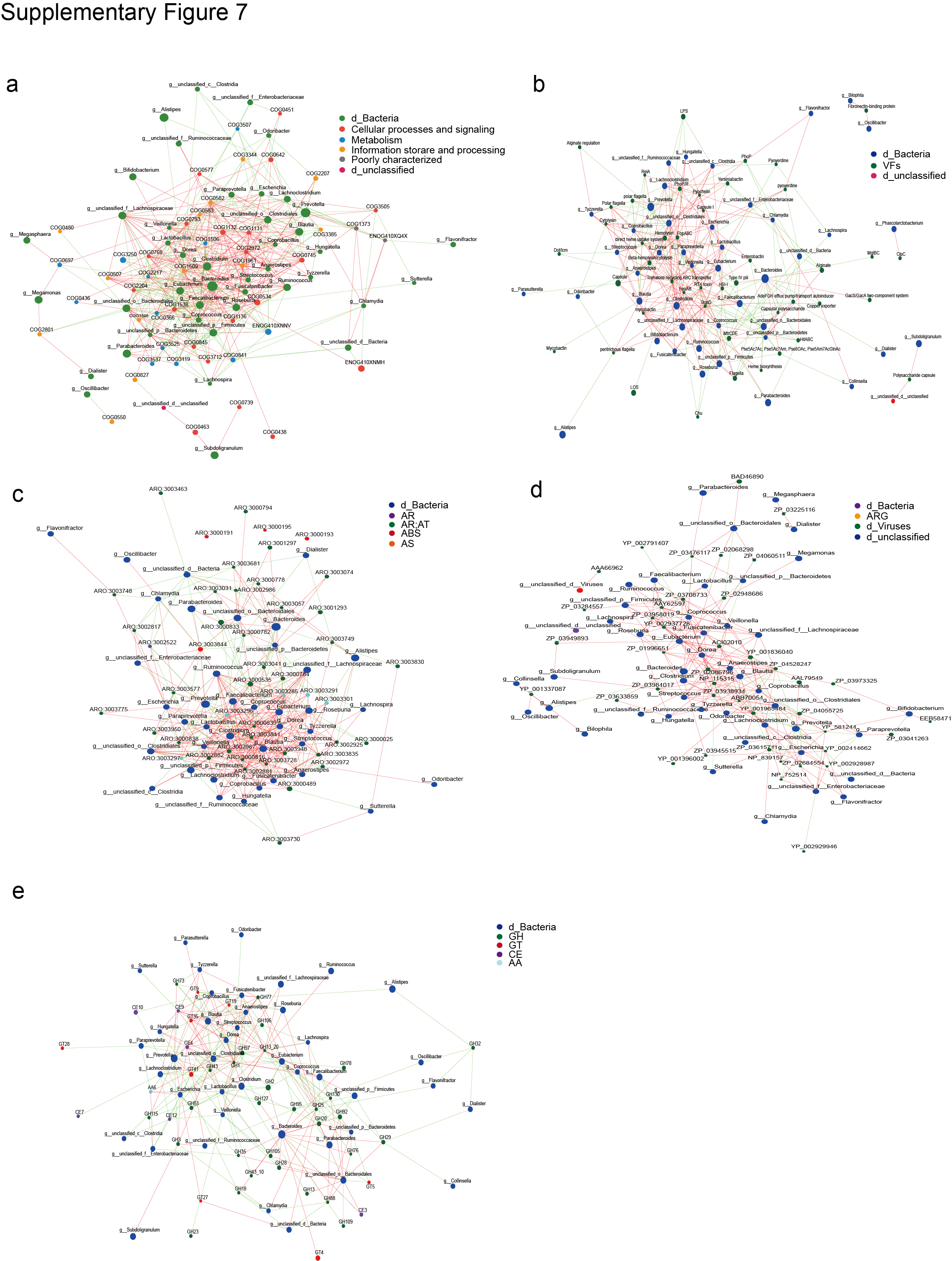

Supplement: Supplementary Figure 7 — (A) The potential relationship between cluster of orthologous groups of proteins (COG) and gut microbiota. (B) The potential relationship between virulent factors and gut microbiota. (C) The potential relationship between antibiotic resistance ontology (ARO) and gut microbiota. (D) The potential relationship between antibiotic resistance genes (ARG) and gut microbiota. (E) The potential relationship between carbohydrate-active enzymes (CAZy) and gut microbiota. [file Image_7.jpeg]

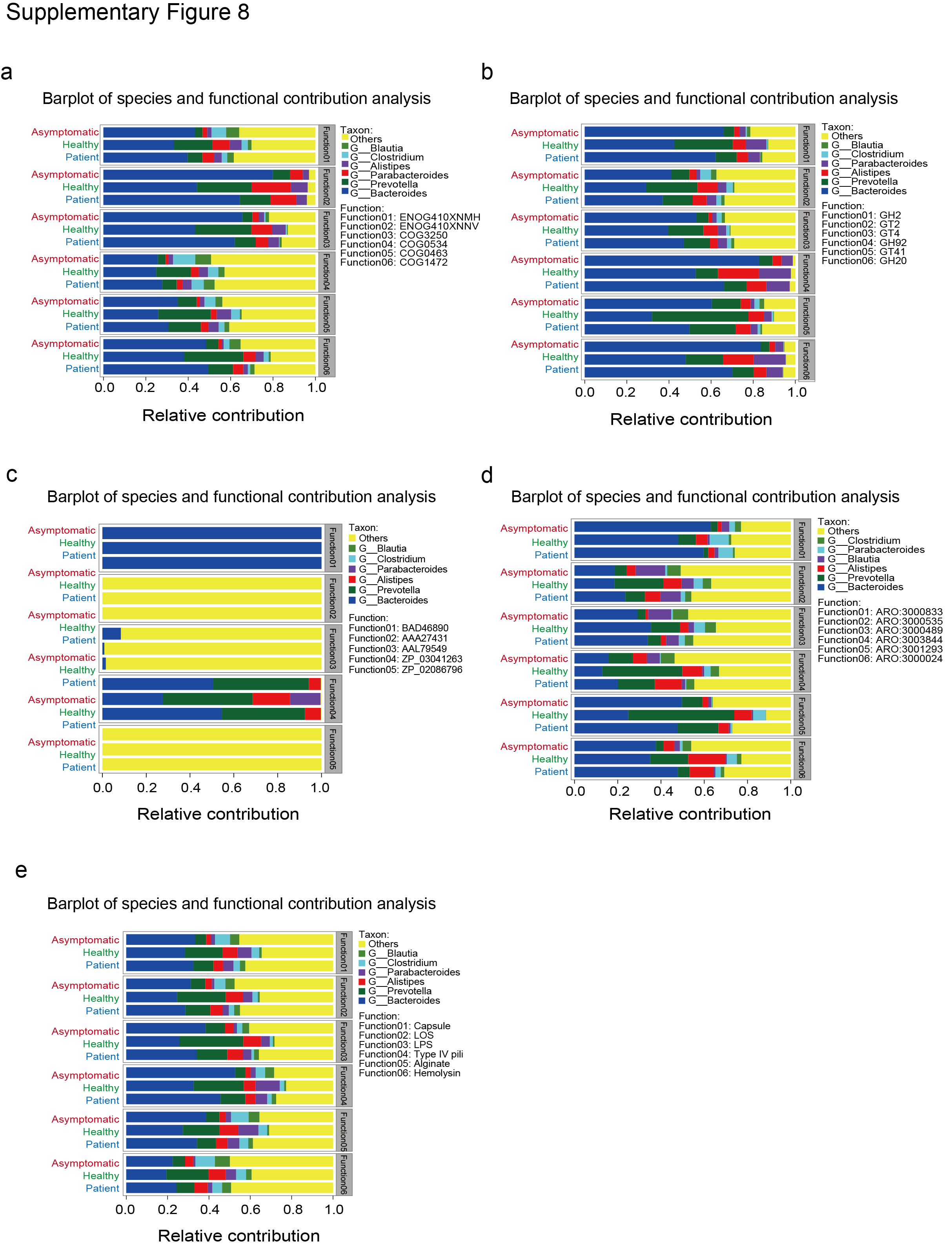

Supplement: Supplementary Figure 8 — (A) Main contributors of species to the functions of cluster of orthologous groups of proteins (COG). (B) Main contributors of species to the functions of carbohydrate-active enzymes (CAZy). (C) Main contributors of species to the functions of antibiotic resistance genes (ARG). (D) Main contributors of species to the functions of antibiotic resistance ontology (ARO). (E) Main contributors of species to the functions of virulent factors. [file Image_8.jpeg]

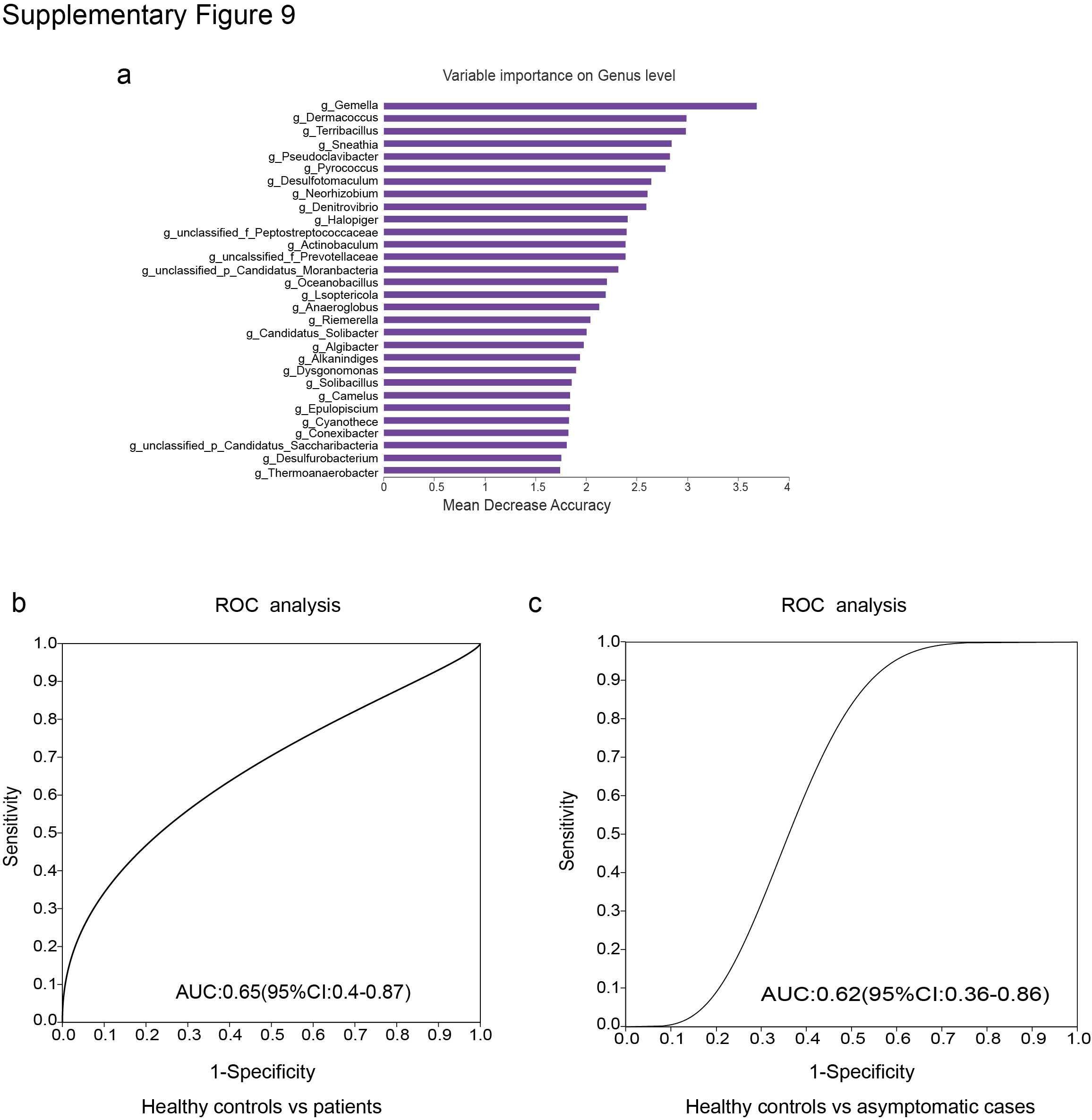

Supplement: Supplementary Figure 9 — (A) The 30 most species identified by the random forest model were shown in rank order of contribution to their prediction accuracy. (B, C) Receiver operating characteristic curves of symptomatic nor asymptomatic cases were not able to be classified successfully. [file Image_9.jpeg]
