# Supplementary material for: Distinct Metagenomic Signatures in the SARS-CoV-2 Infection
Source: Front Cell Infect Microbiol. 2021 Dec 2;11:706970. doi: 10.3389/fcimb.2021.706970 (PMC8674698; doi:10.3389/fcimb.2021.706970)
Supplement: Supplementary file 10 [file Table_1.docx]

**Supplementary Table 1. Baseline characteristics of the participants**

| Indicators/Groups | Healthy (n=10) | Asymptomatic (n=10) | Patient (n=10) | *P*-value |
| --- | --- | --- | --- | --- |
| Age (years) | 39±11 | 36±11 | 44±14 | 0.147 |
| Gender |  |  |  |  |
| Female | 4 (40.0%) | 4 (40.0%) | 3 (30.0%) | 0.878 |
| Male | 6 (60.0%) | 6 (60.0%) | 7 (70.0%) |  |
| Epidemiologic history |  |  |  | 0.211 |
| From Wuhan | - | 0 (0.00%) | 3 (30.0%) |  |
| Close contacts | - | 10 (100.0%) | 7 (70.0%) |  |
