# Supplementary material for: Distinct Metagenomic Signatures in the SARS-CoV-2 Infection
Source: Front Cell Infect Microbiol. 2021 Dec 2;11:706970. doi: 10.3389/fcimb.2021.706970 (PMC8674698; doi:10.3389/fcimb.2021.706970)
Supplement: Supplementary file 11 [file Table_2.docx]

**Supplementary Table 2.** **Generated data of the three groups**

| Indicators/Groups | Healthy (n=10) | Asymptomatic (n=10) | Patient (n=10) | Total | *P*-value |
| --- | --- | --- | --- | --- | --- |
| Read length (bp) | 150 | 150 | 150 | 150 | - |
| Insert size (bp) | 509.2±18.6 | 513.6±16.7 | 495.1±7.9 | 506.0±17.1 | 0.039 |
| Raw reads | 107899024±13106305 | 107573147.6±13674313 | 94573881±11886622 | 103348684±14324835 | 0.060 |
| Raw base (bp) | 16292752714±1979052185 | 16243545288.6±2064821370 | 14280656151±1794880038 | 15605651385±2163050194 | 0.060 |
| Clean reads | 106935004±13152860 | 106505606±13906535 | 93852937±11905344.35 | 102431182±14359646 | 0.070 |
| Clean base (bp) | 16122170430±1983947295 | 16051382285±2101359661 | 14149602676±1802190202 | 15441051797±2168249336 | 0.071 |
| Percent of clean reads in raw reads (%) | 99.09±0.40 | 98.97±0.52 | 99.22±0.25 | 99.09±0.42 | 0.414 |
| Percent of clean reads in raw bases (%) | 98.94±0.40 | 98.77±0.56 | 99.06±0.26 | 98.92±0.44 | 0.357 |
| Optimized reads | 106898417±13139944 | 106140148±14034183 | 93809514±11909689 | 102282693±14369374 | 0.075 |
| Optimized bases (bp) | 16116749298±1982012625 | 15997227153±2120435841 | 14143144441±1802826821 | 15419040297±2169778397 | 0.076 |
| Percent of optimized reads in raw reads (%) | 99.06±0.39 | 98.61±0.99 | 99.18±0.28 | 98.95±0.68 | 0.156 |
| Percent of optimized reads in raw bases (%) | 98.90±0.39 | 98.42±1.06 | 99.02±0.28 | 98.78±0.72 | 0.154 |
