# Supplementary material for: Distinct Metagenomic Signatures in the SARS-CoV-2 Infection
Source: Front Cell Infect Microbiol. 2021 Dec 2;11:706970. doi: 10.3389/fcimb.2021.706970 (PMC8674698; doi:10.3389/fcimb.2021.706970)
Supplement: Supplementary file 12 [file Table_3.docx]

**Supplementary Table 3.** **Predicted genes of the three groups**

| Indicators/Groups | Healthy (n=10) | Asymptomatic (n=10) | Patient (n=10) | Total | *P-*value |
| --- | --- | --- | --- | --- | --- |
| Open reading frames | 288404±77727 | 326943±117721 | 305841±64700 | 307063±90977 | 0.663 |
| Total length (bp) | 184347850±38432123 | 217380770±70916720 | 206712787±41506760 | 206064207±55403800 | 0.667 |
| Average length (bp) | 687±44 | 674±32 | 679±33 | 678±38 | 0.913 |
| Contigs | 147394±51323 | 164055±69515 | 152723±36178 | 154724±54529 | 0.802 |
| Contigs bases (bp) | 223419751±53558966 | 251555234±82702807 | 238351950±47922872 | 237775645±64294167 | 0.645 |
| N50 (bp) | 5720±4882 | 5293±2260 | 4984±2326 | 5332±3397 | 0.899 |
| N90 (bp) | 526±102 | 503±52 | 504±44 | 511±72 | 0.740 |
